# Phenotypic and Genotypic Characteristics of Shiga Toxin-Producing *Escherichia coli* Isolated from Surface Waters and Sediments in a Canadian Urban-Agricultural Landscape

**DOI:** 10.3389/fcimb.2016.00036

**Published:** 2016-04-05

**Authors:** Stephanie Nadya, Pascal Delaquis, Jessica Chen, Kevin Allen, Roger P. Johnson, Kim Ziebell, Chad Laing, Victor Gannon, Susan Bach, Edward Topp

**Affiliations:** ^1^Food, Nutrition and Health, Faculty of Land and Food Systems, University of British ColumbiaVancouver, BC, Canada; ^2^Agriculture and Agri-Food Canada, Summerland Research and Development CentreSummerland, BC, Canada; ^3^Public Health Agency of Canada, National Microbiology Laboratory at GuelphGuelph, ON, Canada; ^4^Laboratory for Foodborne Zoonoses, Public Health Agency of CanadaLethbridge, AB, Canada; ^5^Agriculture and Agri-Food Canada, London Research and Development CentreLondon, ON, Canada

**Keywords:** Shiga toxin-producing *E. coli* (STEC), surface water, sediment, prevalence, whole-genome sequencing

## Abstract

A hydrophobic grid membrane filtration—Shiga toxin immunoblot method was used to examine the prevalence of Shiga toxin-producing *Escherichia coli* (STEC) in four watersheds located in the Lower Mainland of British Columbia, Canada, a region characterized by rapid urbanization and intensive agricultural activity. STEC were recovered from 21.6, 23.2, 19.5, and 9.2% of surface water samples collected monthly from five sites in each watershed over a period of 1 year. Overall prevalence was subject to seasonal variation however, ranging between 13.3% during fall months and 34.3% during winter months. STEC were also recovered from 23.8% of sediment samples collected in one randomly selected site. One hundred distinct STEC isolates distributed among 29 definitive and 4 ambiguous or indeterminate serotypes were recovered from water and sediments, including isolates from Canadian “priority” serogroups O157 (3), O26 (4), O103 (5), and O111 (7). Forty seven isolates were further characterized by analysis of whole genome sequences to detect Shiga toxin gene (*stx 1* and *stx 2)*, intimin gene (*eaeA*) allelic variants and acquired virulence factors. These analyses collectively showed that surface waters from the region support highly diverse STEC populations that include strains with virulence factors commonly associated with human pathotypes. The present work served to characterize the microbiological hazard implied by STEC to support future assessments of risks to public health arising from non-agricultural and agricultural uses of surface water resources in the region.

## Introduction

Shiga toxin-producing *Escherichia coli* (STEC) were formally recognized as agents of human disease in the early 1980s when infections caused by serotype O157:H7 and non-O157 serotypes were definitively linked to watery and bloody diarrheas, thrombotic thrombocytopenic purpura and the hemolytic uremic syndrome (Karmali, 1989; Karmali et al., 2010). Numerous water or food-borne outbreaks have been documented since and persistent rates of community-acquired infections are reported in different continents, countries and regions (Johnson et al., 2006; Gould et al., 2013; Vanaja et al., 2013). A recent analysis of global data suggests that 2,801,000 acute illnesses, 3890 cases of hemolytic uremic syndrome, 270 cases of permanent end-stage renal disease and 230 deaths are attributable to STEC annually (Majowicz et al., 2014). There are more than 400 phenotypically and genotypically diverse STEC serotypes but serotype O157:H7 was long the most commonly reported cause of infections in western countries such as Canada and the United States (Scheutz and Strockbine, 2005; Gill and Gill, 2010). The detection of STEC serotypes other than O157:H7/NM in clinical, food or environmental samples is challenging due to the lack of unique or distinguishing phenotypic features that can be exploited for the differentiation of the group from other *E. coli* strains (Mathusa et al., 2010). Early detection methods primarily targeted *E. coli* O157:H7 because of the apparent epidemiological relevance of this serotype and the relative ease of its detection compared to non-O157 serotypes. The consequent bias in clinical data obscured attempts to determine the historical association between discrete serogroups or serotypes and STEC disease despite some early evidence that testing for non-O157 STEC would identify two-three times more STEC infections than testing only for STEC O157 (Johnson et al., 1996; Stigi et al., 2012). Contemporary improvements in the quality of clinical data stemming from advances in methods for the detection and characterization of STEC have supported more robust estimates of disease causality. It is now apparent that infections with non-O157 serotypes are as frequent or may exceed those attributed to serotype O157:H7 in some jurisdictions (Johnson et al., 2006; Grant et al., 2011; Gould et al., 2013; Vanaja et al., 2013; Byrne et al., 2014; Luna-Gierke et al., 2014). In Canada, for example, slightly more than half of clinical cases reported to the Public Health Agency of Canada surveillance programs are caused by serogroup O157 and the rest are distributed among six additional “priority” serogroups including O26, O103, O111, O117, O121, and O145 (Catford et al., 2014).

Bovines are the most important reservoir of STEC, although other animal species including sheep, horses, deer, goats, pigs, rabbits and birds serve as secondary reservoirs or carriers (Gill and Gill, 2010; Mathusa et al., 2010; Grant et al., 2011). Human exposure may occur by direct means, such as the consumption of contaminated animal products and contact with infected animals or persons, or indirectly following dissemination along variable routes of transmission including contaminated drinking, recreational or irrigation water. Numerous waterborne or fresh produce-associated outbreaks where water likely served as a vector of transmission during crop production have been documented (Muniesa et al., 2006; Getling and Baloch, 2013). There are few reports on the prevalence and characteristics of STEC in surface waters used for home, recreational or agricultural uses despite potential risks to human health. Data on the prevalence of non-O157 STEC in aquatic environments is notably scare. Cooley et al. (2014) recovered both O157 and non-O157 serotypes from surface waters in an agricultural region of the Central California Coast using immunomagnetic bead separation methods. The prevalence of serotype O157:H7 in two successive years was 3.3 and 8%, and that of non-O157 STEC was 14 and 11%. Isolates from non-O157 STEC serogroups O26, O91, O103, and O104 were recovered from water and birds or bird feces within the watersheds examined in this work (Cooley et al., 2014). Non-O157 STEC prevalence and diversity in Canadian surface waters was recently examined by Johnson et al. (2014) in the Grand River of Ontario, a mixed use watershed impacted by point and non-point sources of fecal materials from wildlife, humans and agriculture. A hydrophobic grid membrane filtration—immunoblot method was used to detect and isolate STEC from water samples collected over 2 years. Overall STEC prevalence rates ranged from 11 to 35%; 53 distinct serotypes were recovered from positive water samples, and 37% contained isolates belonging to six of seven priority serogroups in Canada, including O26, O103, O111, O121, O145, and O157. A key finding from this study was that the frequency of O157 and non-O157 STEC isolation and the diversity of STEC recovered far exceeded that achieved in previous surveillance of the watershed using prior analytical approaches (Johnson et al., 2014).

Microbiological hazard characterization and the description of spatio-temporal dynamics affecting their prevalence are imperative for accurate risk assessment and the development of strategies to mitigate transmission and potential human exposure through water. The purpose of the present work was to examine the prevalence, diversity, phenotypic and genotypic characteristics of STEC in surface waters of the Lower Mainland of British Columbia, a densely populated and intensive agricultural region of Canada. This research was carried out with a view to guide future assessment of risks arising from agricultural and non-agricultural uses of regional water resources.

## Materials and methods

### Study location and sampling sites

**Figure 1:**
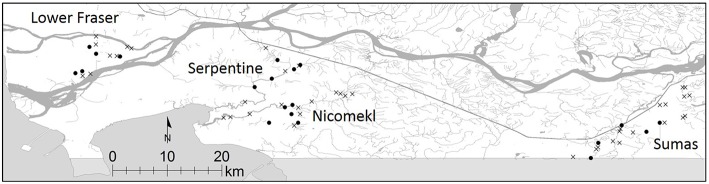
**Location of sampling sites in the Lower Fraser, Sumas, Nicomekl and Serpentine River watersheds of the Lower Mainland of British Columbia, Canada**. Sampling sites selected for the preliminary survey are denoted by x, monthly samplings were carried out in sites are denoted by •.

The study was carried out in the Lower Mainland (LM) of British Columbia, Canada, a broad floodplain extending approximately 130 km east of the city of Vancouver. Duplicate water samples were collected in 61 tributaries, drainage canals and irrigation ditches in four distinct watersheds within the LM (Sumas River, Serpentine River, Nicomekl River and Lower Fraser, Figure 1) between October, 2012 and April, 2013. Additional monthly samplings were conducted between May and November 2013 in five sites selected at random in each watershed. A preliminary assessment of STEC prevalence in sediment was also carried out in one site located on a slow moving stream in the Sumas River watershed. A total of 21 samples were collected from the site in 2012–2013.

### Sample collection

Water samples were collected from each site in sterile 250 ml wide-mouth high density polyethylene bottles (VWR, Edmonton, Canada). The bottles were placed in a holder affixed to the end of a 3 m sampling rod or in a metal bucket attached to a cable, depending on access to the water source. Sediments consisting of a mixture of sand, silt and soft clay were collected by dragging the metal bucket over a distance of approximately 2 m on the surface of the river bed. All samples were kept on ice in a cooler during transport to the laboratory and were held at 4°C prior to analysis.

### Weather data

Mean temperature (T) and precipitation (P) on the day of sampling and 3 days before sampling (Tb, Pb) were obtained from Environment Canada weather stations located in each watershed. Historical weather records for the individual weather stations were retrieved from: http://climate.weather.gc.ca/.

### Detection and isolation of STEC

Detection of STEC in water was accomplished without enrichment using hydrophobic grid membrane filtration—Shiga toxin immunoblot (Stx-IB) methods developed at the Public Health Agency of Canada, National Microbiology Laboratory at Guelph, Canada (PHAC NML), and described by Johnson et al. (2014). All samples were stirred and allowed to settle for 5 min before processing. Supernatants (between 10 and 100 ml, depending on filter performance) were passed through 0.45 μm HGMF filters (Neogen, Lansing, USA) which were incubated at 37°C for 18–24 h on Stx-capture membranes applied to the surface of agar plates containing modified Tryptic Soy Agar (Oxoid, Nepean, Canada) amended with 1.5 g/l bile salts No. 3, 10 ug/ml vancomycin and 10 ug/ml cefsulodin (Sigma, Oakville, Canada) (mTSA-VC). ST-capture membranes consisted of 0.2 um pore size nitrocellulose (Biotrace, Pall Life Sciences, Mississauga, Canada) pre-coated with rabbit anti-ST antibodies reactive to all known Shiga toxins (PHAC NML) and blocked with Phosphate Buffer Saline (PBS)—1% gelatin (Invitrogen, Burlington, Canada; BioRad, Mississauga, Canada). The paired HGMF and Stx-capture membranes on each plate were marked by needle puncture after incubation for later re-orientation. The Stx-capture membranes were removed and probed with a mixture of four monoclonal antibodies (PHAC NML), followed by alkaline phosphatase-labeled rabbit anti-mouse IgG (Jackson Immunoresearch, Cedarlane Laboratories, Burlington, Canada) and the substrate nitroblue tetrazolium and 5-bromo-4-choloro-3-indolyl-phosphate (Sigma). Clearly stained dark purple spots on the Stx-capture membrane denoted the presence of ST. Individual colonies on the HGMF filter corresponding to the location of purple spots on the ST-capture membrane were transferred to either MacConkey agar (Oxoid) or Eosin Methylene Blue (EMB) agar (Oxoid) and incubated at 37°C for 18–24 h for purification. Up to eight isolates were then grown in 500 μl of modified Tryptic Soy Broth (Oxoid) containing 1.0 g/l bile salts No. 3, 10 ug/ml vancomycin and 10 ug/ml cefsulodin (Sigma) (mTSB-VC) in 96-well megablock (Fisher) at 37°C for 18–24 h and the resulting broths were tested to confirm Stx production by ELISA. Confirmation was performed on duplicate 100 μl samples of broth in 96-microwell plates pre-coated with rabbit anti-Stx antibodies (PHAC, NML at Guelph) for 30 min at room temperature. To detect bound Stx, the microwell plates were sequentially incubated for 30 min at room temperature with 100 μl of a mixture of four monoclonal antibodies recognizing all Stx (LFZ), followed by horseradish-peroxidase-labeled rabbit anti-mouse IgG (Jackson Immunoresearch, Cedarlane Laboratories). The wells were washed five times with 300 μl PBS-T after each incubation step, 100 μl of substrate tetramethylbenzidine (Sigma) was added for color development and the plates were incubated with slow agitation for 10 min. The reaction was stopped by addition of 100 μl of 0.2 M sulfuric acid to each well and the mixture was slowly agitated for 10 min. Absorbance was measured immediately with a microplate reader (SpectraMax M2 Microplate Reader, MTX Lab Systems, Inc., US) at a dual wavelength of 450/620 nm. Samples were scored as suspicious or positive for Stx when the mean optical densities (OD) were 1.25–1.5x or >1.5x the mean OD of the negative controls. Controls included a bovine *E. coli* O163:NM strain that produces both Stx1 and Stx2 (strain EC19920459, PHAC NML) and Stx-negative *E. coli* ATCC 25922 as the negative control.

### Confirmation of *E. coli*

The identity of presumptive isolates was confirmed using a monoplex-PCR assay targeting the *E. coli gadA* gene according to methods described in Doumith et al. (2012). Individual test cultures were grown at 37°C overnight in 2.5 ml TSB (Oxoid). An aliquot (360 μl) of the culture was transferred to a 1.5 ml microcentrifuge tube (Invitrogen) with 40 μl 10X pH 7.2 PBS (Invitrogen). The mixture was heated at 96°C under constant agitation at 600 rpm for 10 min. After heating, the microcentrifuge tube was placed on ice for 10 min and was spun in a centrifuge (Microcentrifuge 5415 R, Eppendorf, Mississauga, Canada) at 13,200 rpm for 5 min. The supernatant containing DNA lysate was decanted and stored at −20°C until analyzed. PCR was performed with 1 μl DNA lysate amplified with TopTaq DNA Polymerase (Qiagen, Canada) in 25 μl reaction mixtures containing 1X Buffer Solution, 1X Coral Dye, 50 μM dNTP's (Invitrogen), 0.625 U/rxn TopTaq DNA Polymerase, 5 μl Q-solution and 1 μM of the primers:

1. gadA-F 5′-GATGAAATGGCGTTGGCAAG-3′;
2. *gadA-R 5′-GGCGGAAGTCCCAGACGATATCC-3′*.

The PCR reaction was carried out under the following conditions in a thermal cycler (C1000 Touch Thermal Cycler, BioRad, Canada): 94°C for 4 min, followed by 30 cycles consisting of 94°C for 30 s, 65°C for 30 s and 72°C for 30 s, with a final extension step at 72°C for 5 min. PCR products were held at 4°C until they could be visualized in SYBR^®;^ Safe (Invitrogen, Canada) stained 2% agarose gels following electrophoresis using 1X TAE buffer (BioRad) at 80V for 45 min. The expected amplicon size for *gadA* was 373 bp. A clinical *E. coli* O157:H7 strain graciously provided by Linda Hoang (BC Centre for Disease Control, Vancouver, British Columbia, Canada) was used as positive control for *gadA* and water was used as negative control.

### Serotyping

Isolates confirmed as *E. coli* were submitted to the *E. coli* Reference Laboratory (PHAC NML at Guelph) for serotyping. Somatic (O) and flagellar (H) antigens were identified by accredited methods using reference antisera (SSI Diagnostica, Copenhagen, Denmark). Flagellar antigens were identified after 2–7 days incubation in 0.28% motility agar at 37°C, and if necessary, in 0.25% motility agar for 1–7 days at 37°C and 1–7 days at 20–22°C. Isolates not exhibiting motility after this time were designated non-motile (NM).

### Fingerprinting by Rep-PCR

A Rep-PCR fingerprinting technique was used to differentiate isolates where several were recovered from the same water sample. Rep-PCR was performed using the BOX A1R primer (5′-CTACGGCAAGGCGACGCTGACG-3′) according to methods described in Dombek et al. (2000). Template DNA was extracted with a Qiagen DNeasy Blood & Tissue Kit (Qiagen, Canada) from 1.5 mL of and overnight culture grown at 37°C in Tryptic Soy Broth. Five microliters of DNA template from each isolate was amplified in 25 μl reaction mixtures containing 12.5 μl Multiplex PCR Master Mix (Qiagen), 1.4 μM BOX A1R primer and 2.5 Q-solution. The PCR reaction was carried out in a thermal cycler (BioRad) programmed to provided 95°C for 2 min, followed by 35 cycles of 94°C for 3 s, 92°C for 30 s, 50°C for 1 min and 65°C for 8 min, and a final extension step of 65°C for 8 min. PCR products were visualized in SYBR^®;^ Safe (Invitrogen) stained 1.5% agarose gels following electrophoresis using 1X TAE buffer (BioRad) at 50 V for 900 min in a cold room at 4°C. After electrophoresis, the gel was further stained in SYBR® Safe in 1X TAE buffer (1:50 ratio) for 30 min with gentle agitation before imaging.

### Virulence gene profiling by PCR

The presence of virulence genes *eaeA, hlyA, stx*_1_, and *stx*_2_ was verified by multiplex PCR according to methods described by Paton and Paton (1998). Template DNA was prepared by the boiling extraction method described above. DNA lysate (1 μl) from each isolate was amplified with TopTaq DNA Polymerase (Qiagen) in 25 μl reaction mixtures containing 1X Buffer Solution, 1X Coral Dye, 50 μM dNTP's (Invitrogen), 0.625 U/rxn TopTaq DNA Polymerase, 5 μl Q-solution, and 1 μM of *eae* and *hlyA* primers, and 0.4 μM of *stx*_1_ and *stx*_2_ primers. The PCR reaction was carried out under the following conditions in a thermal cycler (Biorad): 95°C for 3 min, followed by 35 cycles each consisting of a denaturation step at 95°C for 1 min; an annealing step at 65°C for 2 min for the first 10 cycles, decrementing 1°C per cycle to 60°C by cycle 15; and an elongation step at 72°C for 1.5 min, incrementing to 2.5 min from cycles 25–35; and a final extension step at 72°C for 5 min. PCR products were visualized in SYBR^®;^ Safe (Invitrogen) stained 2% agarose gels following electrophoresis using 1X TAE buffer (BioRad) at 80 V for 45 min. The clinical *E. coli* O157:H7 isolate (Strain ID, BC Centre for Disease Control, *stx*_1_, *stx*_2_, *eaeA, hlyA* positive)was used to verify PCR assay performance.

### Genome sequencing and assembly

Genomic DNA was isolated from overnight cultures grown in TSB (Oxoid) at 37°C using a MasterPure DNA Purification Kit. (Epicentre, Madison, WI). DNA was subjected to picogreen quantitation (Quant-iT^TM^ PicoGreen, ThermoFisher Scientific, Waltham, MA) prior to library preparation and Illumina sequencing. Briefly, genomic DNA libraries were prepared using a NexteraXT kit according to manufacturer's instructions and up to 12 libraries were pooled on an individual flow cell and subjected to paired-end 250 bp sequencing on an Illumina Miseq (Illumina, San Diego, CA). Adapter sequences were removed using FastX clipper v1.0.1 and low quality bases were removed from raw sequencing reads using FastQ quality trimmer v1.0.0 from the Hannon Lab FastX tool kit v 0.0.13 in Galaxy (http://hannonlab.cshl.edu/fastx_toolkit/index.html). Reads were assembled *de novo* using SPAdes v. 3.1 (Bankevich et al., 2012) to generate draft sequence assemblies that were subsequently annotated using Prokka v. 1.10 (Seemann, 2014). Annotations were guided by converting the Virulence Factor Database (VFDB) and using a trusted annotation file within Prokka to ensure proper annotation of *E. coli* virulence factors (Chen et al., 2012). Genome assembly quality metrics were calculated using QUAST v. 2.3 (Gurevich et al., 2013).

### Genomic analyses

A core genome single nucleotide polymorphism (SNP) phylogeny was generated using Parsnp v1.2 (Treangen et al., 2014) with EDL933 as a reference and while employing the –x option to omit bases identified to have undergone recombination. Output from Parsnp is an approximately maximum likelihood tree with local support values ranging from 0 to 1 based on 1000 resamples and the Shimodaira Hasegawa test produced in FastTree2 (Price et al., 2010). Genomes for the following clinical isolates from various STEC serogroups were also included for comparison (serotype, strain number (Genbank assembly accession number)): O103:H11, 2010C-3214 (GCA_000615055.1); O103:H2, 12009 (GCA_000010745.1); O103:H25, NIPH-11060424 (GCA_000234605.2); O104:H4, 2009EL-2050 (GCA_000299255.1); O104:H4, 2011C-3493 (GCA_000299455.1); O111:H8, 2009C-4126 (GCA_000632635.1); O111:NM, 01-3076 (GCA_000701125.2); O111:NM, 2009C-4006 (GCA_000619225.2); O165:H25, 2010C-4874 (GCA_000617585.1); O26:H11, 03-3500 (GCA_000622445.2); O26:H11, 11368 (GCA_000091005.1); O26:NM, 2010C-4347 (GCA_000614845.2); O157:H7, Sakai, (GCA_000008865.1).

Shiga toxin gene subtyping was performed using a previously described BLASTn based approach (Ashton et al., 2015) using Blast+ v2.2.29. Briefly, assemblies were queried for the *stx1* and *stx2* genes using the *stx* gene reference set and an *E*-value cutoff of 1 × 10^−20^ as described by Ashton et al. (2015). Subtypes were assigned according to the match yielding the highest bit score. Intimin gene (*eae*) variants were assigned using a BLASTn based approach modeled after the approach outlined above for the *stx* genes. At least 24 intimin variants have been described to date, although described nomenclature varies by publication. The following reference sequences and nomenclature were used to identify and assign intimin variants in genome assemblies: *eae*-α1 (M58154.1), *eae*-α2 (AF530555.1), *eae*-β1 (AF200363.1), *eae*-β2 (AF530556.1), *eae*-γ (AF071034.1), *eae*-δ (AJ875027.1), *eae*-ε1 (AF116899.1), *eae*-ε2 (AF530554.1), *eae*-ζ (AJ271407.1), *eae*-η1 (AJ308550.1), *eae*-η2 (AJ876652.1), *eae*-θ (AF449418.1), *eae*-ι1 (AJ308551.1), *eae*-ι2 (AF530553.1), *eae*-κ (AJ308552.1), *eae*-λ (AJ715409.1), *eae*-μ (AJ705049.1), *eae*-ν (AJ705050.1), *eae*-o (AJ876647.1), *eae*-π (AJ705052.1), *eae*-ρ (EF204930.1), *eae*-σ (AJ781125.1), *eae*-τ (FM872416.1), *eae*-υ (FM872417.1), *eae*-χ (AJ705051.1). *In silico* serotypes were also determined with SerotypeFinder v. 1.1 (https://cge.cbs.dtu.dk/services/SerotypeFinder/) using default parameters (Joensen et al., 2015). The *E. coli* specific Virulence Finder database (virulence_ecoli.fsa; 04-Jan-2016 version) was downloaded from the Center for Genomic Epidemiology website and used to query the 47 genomes assemblies from this study, and the 15 *E. coli* reference genomes using BLASTn (blast+ v2.3.0). A gene was considered “present” in a genome if any allele in the virulence factor database was present with at least 72% total sequence identity (90% identity over 80% of the gene). The results were visualized in conjunction with the SNP phylogeny using the R package “ggtree” (https://github.com/GuangchuangYu/ggtree) in the script “tree_matrix.R.” Illumina reads and metadata generated in this study will be submitted to the NCBI Sequence Read Archive (SRA; http://www.ncbi.nlm.nih.gov/sra/) under umbrella Bioproject SRP PRJNA287560.

### Statistical analyses

Prevalence in each region and for different seasons was calculated from the number of samples from which STEC was isolated divided by the total number analyzed. The Chi square (χ^2^) test was applied to compare prevalence between seasons. Relationships between average temperature (T) and total precipitation (P) on the day of sampling, average temperature 3 days before sampling (Tb) and cumulative precipitation 3 days before sampling (Pb) and STEC prevalence were first examined using a point biserial correlation. This approach was selected because the variables were dichotomous for the presence and absence of STEC and continuous for the environmental factors (Gu et al., 2013). All analyses were performed with the R software package (R Core Development Team, Vienna, Austria). The probability of significance *p* was ≤ 0.05 unless otherwise specified.

## Results

### STEC prevalence in surface waters and sediments

STEC were recovered from 20.3% of surface water samples collected in 61 sites in the Sumas, Serpentine and Nicomekl River watersheds during a preliminary survey carried out between October, 2012 and April, 2013, but not from waters in the Lower Fraser watershed. Additional samples were collected monthly until November 2013 in five sites in each watershed. Results provided in Table 1 show that overall STEC prevalence rates were 23.2, 21.6, and 19.5% in the Serpentine, Sumas, Nicomekl River watersheds respectively, and 9.2% in the Lower Fraser watershed. STEC were also recovered from 23.8% of 21 sediment samples collected in a single site located in the Sumas River watershed site (Table 1). However, analysis using the Fisher exact test revealed a low probability (*P* = 0.573) of simultaneous STEC detection in water and sediment at the site.

**Table 1:** **Numbers of water and sediment samples analyzed and STEC prevalence rates in four watersheds of the Lower Mainland of British Columbia between November 2012 and November 2013**.

**Watershed**	**No. of samples analyzed**	**Prevalence rate (%)**
**WATER**		
Lower Fraser	65	9.2
Sumas River	97	21.6
Nicomekl River	86	23.2
Serpentine River	82	19.5
	Total: 330	Overall: 19.1
**SEDIMENTS**		
Sumas	21	23.8

*Water samples were collected monthly in 5 sampling sites in each watershed, sediment samples on 5 separate occasions over a period of 1 year*.

**Figure 2:**
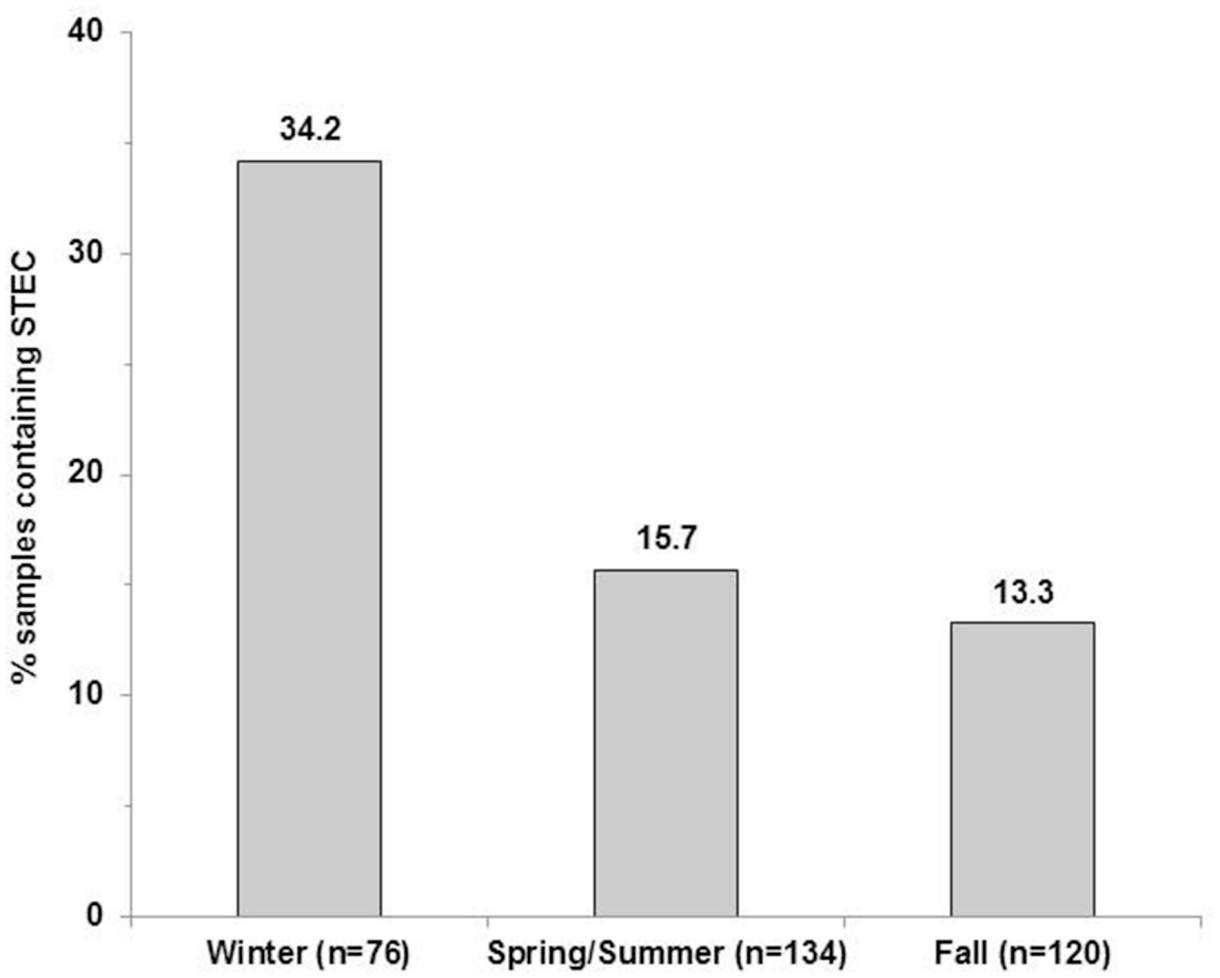
**STEC prevalence in surface water samples collected in the Lower Fraser, Sumas, Nicomekl and Serpentine River watersheds of the Lower Mainland of British Columbia, Canada**. Columns show the proportion of surface water samples (%) from which STEC were recovered between December 2012–February 2013(winter), May 2013–August 2013 (spring/summer) and September–November 2013 (fall).

The LM pacific coastal ecoregion is characterized by cool temperatures and high annual precipitation, primarily in the form of rain. Historical climate data for each watershed (Table 2) showed that precipitation is highest during the winter (December–February), intermediate in the fall (September–November) and comparatively slight during spring and summer (May–August) months. A graphical representation of STEC prevalence on a seasonal basis (Figure 2) revealed that prevalence was highest during winter months, approaching 35% in surface water samples collected in all watersheds. The influence of climate was further examined by analysis of correlation between temperature and precipitation on or 3 days before sampling and recovery from the samples. Temperature and average precipitation 3 days before sampling were significantly correlated (*p* < 0.05) with the presence of STEC in water when the data were pooled (Table 3). The probability of correlation varied between watersheds, however, and neither factor was correlated with STEC recovery from the Nicomekl watershed. Moreover, attempts to correlate prevalence with sampling location were unsuccessful due to large and random variation in the frequency of recovery from discrete sites.

**Table 2:** **Average monthly temperatures and precipitation in four watersheds of the Lower Mainland of British Columbia**.

**Watershed**	**Month**											
	**J**	**F**	**M**	**A**	**M**	**J**	**J**	**A**	**S**	**O**	**N**	**D**
**AVERAGE TEMPERATURE(°C)**												
Sumas River	2.3	4.7	7.0	10.2	13.4	15.9	18.5	18.4	15.7	10.8	6.1	3.1
Serpentine/Nicomekl	3.7	5.4	7.6	10.6	13.8	16.2	18.3	18.5	15.9	11.0	6.5	3.4
Lower Fraser	3.4	5.0	7.3	9.9	13.1	15.8	18.0	18.0	14.9	10.3	5.8	3.2
**AVERAGE PRECIPITATION (mm)**												
Sumas River	225	166	170	136	112	93	61	55	89	172	258	239
Serpentine/Nicomekl	196	118	112	100	85	64	50	37	61	142	201	188
Lower Fraser	164	132	111	94	74	66	39	44	62	120	197	175

**Table 3:** **Correlation of temperature and precipitation with STEC recovery from surface waters in the Lower Mainland of British Columbia**.

**Environmental factor^a^**	**Correlation with STEC occurrence**	***p*^b^**
**LOWER FRASER**		
T	−0.206	0.008^*^
T_b_	−0.239	0.002^*^
P	0.056	0.475
P_b_	0.187	0.017^*^
**SUMAS**		
T	−0.387	0.005^*^
T_b_	−0.391	0.005^*^
P	−0.023	0.875
P_b_	0.276	0.052
**NICOMEKL**		
T	−0.015	0.925
T_b_	−0.009	0.957
P	−0.062	0.684
P_b_	−0.018	0.908
**SERPENTINE**		
T	−0.415	0.007^*^
T_b_	−0.353	0.023^*^
P	0.106	0.509
P_b_	0.078	0.626
**ALL DATA**		
T	−0.194	0.011^*^
T_b_	−0.218	0.004^*^
P	0.062	0.421
P_b_	0.196	0.010^*^

*Results of analyses performed for all data and that collected in Lower Fraser, Sumas, Nicomekl and Serpentine River watersheds are shown*.

a Environmental factors: T, Mean temperature (°C) on the day of sampling; T_b_, Mean temperature (°C) for 3 days before sampling; P, Precipitation accumulation (mm) on the day of sampling;

P_b_, Cumulative precipitation (mm) for 3 days before sampling;

b *Probability of correlation between the environmental factor and STEC occurrence*.

* *Denotes significance (p < 0.05)*.

### Phenotypic and genotypic characterization of STEC isolates

Presumptive STEC isolates recovered by the hydrophobic grid membrane filtration—ST immunoblot method varied in numbers ranging from 1 to >40 per sample. Comparison of REP-PCR generated genomic fingerprints, virulence gene profile by PCR, confirmation of ST production by ELISA and serological analysis led to the recognition of 100 ostensibly unique isolates distributed among 29 definitive and 4 ambiguous or indeterminate serotypes, including 3 isolates from Canadian “priority” serogroup O157, 4 from O26, 5 from O103 and 7 from O111 (Table 4). Virulence gene *stx1* was detected by PCR in 83%, *stx2* in 53%, both *stx1* and *stx2* in 35%, *eaeA* in 39%*, hlyA* in 64%, and all four *stx1, stx2, eaeA*, and *hlyA* genes in 10% of the isolates. Some serogroups were recurrent (e.g., O111), while others were isolated infrequently (e.g., O8, O116, O168, O177). It must be noted here that isolates derived from samples with high recovery rates were occasionally assigned identical serological assignment despite apparent differences in fingerprints derived from REP-PCR. However, low resolution or variable banding patterns on agarose gels (data not shown) often introduced uncertainty that prevented clear differentiation between distinct and clonal isolates. Consequently, whole genome sequence (WGS) analyses were carried out to differentiate serologically analogous isolates. Randomly selected isolates from other serogroups and one reference strain were also sequenced (total = 48) with a view to examine genotypic diversity in STEC from the region.

**Table 4:** **Virulence gene profiles determined by PCR and conventional serological assignments of STEC recovered from surface waters and sediments in the Lower Mainland of British Columbia (*n* = 100)**.

**Virulence gene profiles**	**Serotypes**
*stx1, stx2,eaeA, hlyA (9)*	O111:H8 (1), O111:NM (2), O157:H7 (2), O157:NM (1), O165:H25 (1), O165:NM (2)
*stx1, stx2, hlyA (22)*	O8:H19 (2), O88:H25 (5), O128:H2 (3), O151:H12 (1), O163:NM (3), O163:H19 (4), OR:NM (3), O?:H19 (1)
*stx1, stx2 (4)*	O91:NM (1), O128:H2 (2), O174:H8 (1)
*stx1, eaeA, hlyA (26)*	O5:NM (2), O26:H11 (4), O69:H11 (1), O84:H2 (2), O98:NM (2), O103:H2 (5), O103:H11 (1), O103:H25 (3), O111:H8 (3), O111:NM (1), O156:H25 (2)
*stx1, eaeA (1)*	O69:H11 (1)
*stx1,hlyA (3)*	O76:H19 (2), O136:H12 (1)
*stx1 (18)*	O6:H10 (1), O136:H12 (4), O136:H16 (1), O146:H8 (3), O182(O109):H5 (8)
*stx2, eaeA, hlyA (3)*	O165:NM (2), O177:NM (1)
*stx2, hlyA (2)*	O8:H19 (2)
*stx2 (12)*	O8:H9 (1), O22:H8 (1), O113:H21 (1), O116:H25 (3), O130:H8 (1), O141ac:H8 (1), O168:H8 (1), O174:H21 (2), OR:H21 (1)

*The number of isolates for individual profiles or serotypes is provided in brackets*.

**Figure 3:**
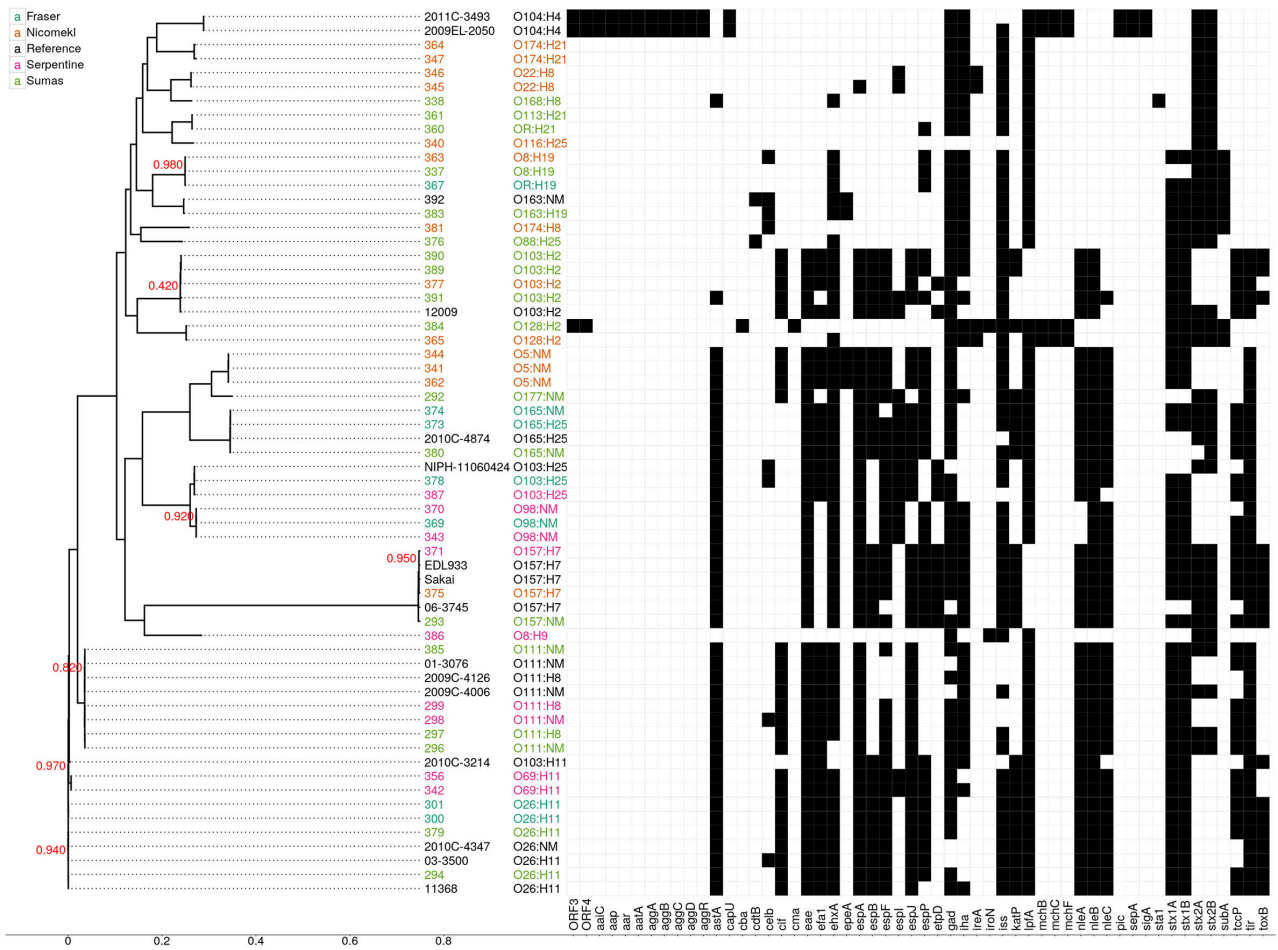
**Approximate maximum likelihood tree and presence/absence matrix for 54 acquired virulence factor genes detected in 63 STEC genomes, including 48 from the present study and 15 reference genomes from clinical isolates**. The phylogeny was generated using Parsnp with EDL933 as a reference. Node labels indicate local support values (range from 0 to 1) based on 1000 resamplings and the Shimodaira Hasegawa test in FastTree2. The tree was based on 118,086 core SNP loci and the scale corresponds to the number of substitutions per SNP. Isolate numbers and serotypes are assigned different colors according to source as shown in the legend. Black squares in the virulence factor matrix indicate the presence of a virulence gene in the Virulence Factor Database at a sequence identity of at least 72%, while white indicates the absence of the gene. Reference genomes: O103:H11 (2010C-3214), O103:H2 (12009), O103:H25 (NIPH-11060424), O104:H4 (2009EL-2050), O104:H4 (2011C-3493), O111:H8 (2009C-4126), O111:NM (01-3076), O111:NM (2009C-4006), O165:H25 (2010C-4874), O26:H11 (03-3500), O26:H1 (11368), O26:NM (2010C-4347), O157:H7 (EDL933), O157:H7 (Sakai). Virulence factor genes and their associated functions: ORF 3 and 4, open reading frame, O42 plasmid; aaiC, Secreted protein of EAEC; aap, dispersin-encoding gene; aar, AggR-activated regulator; aatA, pAA virulence plasmid marker gene; aggA, Aggregative adherence fimbriae I; aggB, protein aggB precursor; aggC, Outer membrane usher protein; aggD, Chaperone protein; aggR, Transcriptional activator; astA, EAST-1 heat-stable toxin; cap U, Hexosyltransferase homolog; cba, Colicin B; cdtB, Cytolethal distending toxin B; celb, Endonuclease colicin E2; cif, Type III secreted effector; cma, Colicin M; efa1, EHEC factor for adherence; eae, intimin adherence protein; efa1, Elongation factor 1-alpha; ehxA, Enterohaemolysin; epeA, Autotransporter protease; espA, Type III secretion system; espB, Secreted protein B; espF, Type III secretion system; espI, Serine protease autotransporters of Enterobacteriaceae (SPATE); espJ, Prophage-encoded type III secretion system effector; espP, Extracellular serine protease plasmid-encoded; etpD, Type II secretion protein; gad, Glutamate decarboxylase: iha, Adherence protein; ireA, Siderophore receptor; iroN, Enterobactin siderophore receptor protein; iss, Increased serum survival; katP, Plasmid-encoded catalase peroxidase; lpfA, Long polar fimbriae; mchB, Microcin H47 part of colicin H; mchC, mch C protein; mchF, ABC transporter protein; nleA, Non-LEE encoded effector A; nleB, Non-LEE encoded effector B, nleC: Non-LEE encoded effector C; pic, Protein involved in intestinal colonization; sepA, Serine protease A precursor; sigA, serine protease A; sta1, Heat-stabile enterotoxin ST-IA; stx1A, Shiga- like toxin 1 subunit A; stx 1B, Shiga- like toxin 1 subunit B; stx2A, Shiga- like toxin 2 subunit A; stx2B, Shiga- like toxin 2 subunit B; subA, Subtilase toxin subunit; tccP, Tir-cytoskeleton coupling protein; tir, Translocated intimin receptor protein; toxB, Toxin B.

Draft genome sequencing of the 47 isolates and 1 reference strain produced assemblies with a median number of contigs of 198 (range: 59–401) and a median N50 value of 112,973 (range: 78,678–242,732). An approximate maximum likelihood tree derived from analysis of genome-wide SNPs is shown in Figure 3, wherein each isolate is designated by a three digit number for linkage to relevant sample data and additional genotypic characteristics deduced from WGS analyses (see Table 5 below). Six unique isolates (292-O177:NM, 338-O168:H8, 340-O116:H25, 376-O88:H25, 381-O174:H8, 386-O8:H9) were positioned on individual branches of the tree. The balance was assigned to clusters consisting of isolates with identical serology and occasionally contradictory O or H antigen types. O and H antigen types were predicted from genome assemblies to validate assignments and to establish the basis for serological differences within some of the clusters. Results presented in Table 5 showed that O and H types determined from conventional and WGS-based serology were identical in 30 of the 48 isolates. Fourteen of the remaining 18 isolates were assigned an *in silico* H type despite their non-motile phenotype. The latter was not unexpected given the reported prevalence of H antigen genes or gene variants in non-motile STEC (Joensen et al., 2015). Genes for O antigens may likewise be detected in strains that cannot be assigned an O-type by conventional analysis, as illustrated by isolates 360-OR:H21 (predicted type O113) which displayed a rough (R) phenotype and 367-O?:H19 (predicted O type O8) that did not react with commercial antisera. Moreover, the O128abc antiserum employed in the present work did not allow differentiation of type O128 and sub-group O128ab and O128ac strains derived from O-antigen processing system gene variants (Joensen et al., 2015). Hence, conventional and *in silico* serotyping data confirmed that isolates within clusters defined by parsimony analysis of genome-wide SNPs were antigenically homologous and likely derived from common lineages. It was interesting to note that O26:H11 and O69:H11 isolates situated in two proximal clusters on a common node of the tree were of identical *stx* and *eaeA* gene subtypes and shared acquired virulence factor genes (see below). Previous phylogenetic studies based on multilocus sequence typing analysis of seven housekeeping genes (Ziebell et al., 2008) and genome-wide SNPs (Ju et al., 2012) have also suggested that the two serotypes are closely related.

**Table 5:** **Predicted serotypes, *stx* and *eaeA* gene subtypes derived from whole genome sequence analysis of 47 STEC isolates recovered from surface waters and sediments in different sampling sites within four watersheds of the Lower Mainland of British Columbia. Isolates with similar serotypes and their origin are also shown**.

**Isolate number and serotype**	**Predicted serotype**	**Watershed**	**Source**	**Site**	***stx gene subtype***	***eaeA gene subtype***	**Isolates with similar serotypes and their origin**			
							**Sumas**	**Nicomekl**	**Serpentine**	**Fraser**
360-OR:H21	O113:H21^*^	Sumas	Water	1	*stx2_*d*_*	NA^a^	361			
384-O128:H2	O128ab:H2^*^	Sumas	Water	1	*stx1_*c*_ + stx2_*b*_*	NA		365		
337-O8:H19	O8:H19	Sumas	Water	2	Mult.? *stx2_*a*_, stx2_*d*_*^b^	NA		363		367
294-O26:H11	O26:H11	Sumas	Water	2	*stx1_*a*_*	β1	379 (49)^c^			300, 301
379-O26:H11	O26:H11	Sumas	Water	2	*stx1_*a*_*	β1	294 (49)			300, 302
385-O111:NM	O111:H8^*^	Sumas	Water	2	*stx1_*a*_ + stx2_*a*_*	θ	296, 297		298, 299	
293-O157:NM	O157:H7^*^	Sumas	Water	2	*stx1_*a*_+ stx2_*c*_*	γ		375	371	
380-O165:NM	O165:H25^*^	Sumas	Water	2	Mult.? *stx2_*c*_, stx2_*a*_*	ε				373, 374
292-O177:NM	O177:H25^*^	Sumas	Water	2	*stx2_*c*_*	β1				
383-O163:H19	O163:H19	Sumas	Water	3	*stx1_*a*_ + stx2_*a*_*	NA				
376-O88:H25	O88:H25	Sumas	Water	4	*stx1_*a*_+ stx2_*a*_*	NA				
389-O103:H2	O103:H2	Sumas	Sediment	4	*stx1_*a*_*	ε	390, 391	377		
390-O103:H2	O103:H2	Sumas	Sediment	4	*stx1_*a*_*	ε	389, 391 (14)	377		
391-O103:H2	O103:H2	Sumas	Sediment	4	*stx1_*a*_*	ε	389 (14), 390 (14)	377		
296-O111:NM	O111:H8^*^	Sumas	Water	4	*stx1_*a*_+ stx2_*a*_*	θ	297, 385		298, 299	
297-O111:H8	O111:H8	Sumas	Water	4	*stx1_*a*_ + stx2_*a*_*	θ	296, 385		298, 299	
361-O113:H21	O113:H21	Sumas	Water	4	*stx2_*d*_*	NA	360			
338-O168:H8	O168:H8	Sumas	Water	4	*stx2_*a*_*	NA				
386-O8:H9	O8:H9	Serpentine	Water	6	*stx2_*d*_*	NA				
342-O69:H11	O69:H11	Serpentine	Water	6	*stx1_*a*_*	β1			342 (37)	
356-O69:H11	O69:H11	Serpentine	Water	6	*stx1_*a*_*	β1			356 (37)	
343-O98:NM	O98:H21^*^	Serpentine	Water	6	*stx1_*a*_*	ζ			369 (141), 370	
369-O98:NM	O98:H21^*^	Serpentine	Water	6	*stx1_*a*_*	ζ			343 (141), 370	
370-O98:NM	O98:H21^*^	Serpentine	Water	6	*stx1_*a*_*	ζ			369, 343 (141)	
298-O111:NM	O111:H8^*^	Serpentine	Water	6	*stx1_*a*_*	θ	296, 297, 385		299	
299-O111:H8	O111:H8	Serpentine	Water	6	*stx1_*a*_*	θ	296, 297, 385		298	
371-O157:H7	O157:H7	Serpentine	Water	6	*stx1_*a*_ + stx2_*a*_*	γ	293	375		
387-O103:H25	O103:H25	Serpentine	Water	8	*stx1_*a*_*	θ				378
341-O5:NM	O5:H9^*^	Nicomekl	Water	10	*stx1_*a*_*	β1		344 (35), 362		
344-O5:NM	O5:H9^*^	Nicomekl	Water	10	*stx1_*a*_*	β1		341 (35), 362		
340-O116:H25	O116:H25	Nicomekl	Water	10	*stx2_*d*_*	NA				
365-O128:H2	O128ac:H2^*^	Nicomekl	Water	10	*stx1_*c*_ + stx2_*b*_*	NA	384			
375-O157:H7	O157:H7	Nicomekl	Water	10	*stx1_*a*_ + stx2_*a*_*	γ	293		371	
381-O174:H8	O174:H8	Nicomekl	Water	10	*stx1_*c*_+ stx2_*b*_*	NA				
362-O5:NM	O5:H9^*^	Nicomekl	Water	11	*stx1_*a*_*	β1		341, 344		
363-O8:H19	O8:H19	Nicomekl	Water	11	*stx1_*a*_ + stx2_*a*_*	NA	337			367
345-O22:H8	O22:H8	Nicomekl	Water	11	*stx2_*d*_*	NA		346		
346-O22:H8	O22:H8	Nicomekl	Water	11	*stx2_*d*_*	NA		345		
377-O103:H2	O103:H2	Nicomekl	Water	11	*stx1_*a*_*	ε	389, 390, 391			
364-O174:H21	O174:H21	Nicomekl	Water	11	*stx2_*a*_*	NA		347		
347-O174:H21	O174:H21	Nicomekl	Water	12	*stx2_*a*_*	NA	364			
300-O26:H11	O26:H11	Fraser	Water	13	*stx1_*a*_*	β1	294, 379			301
373-O165:H25	O165:H25	Fraser	Water	13	*stx1_*a*_ + stx2_*a*_*	ε	380			374
374-O165:NM	O165:H25^*^	Fraser	Water	13	*stx1_*a*_ + stx2_*a*_*	ε	380			373
367-O?:H19	O8:H19^*^	Fraser	Water	14	*stx1_*a*_ + stx2_*a*_*	NA	337	363		
301-O26:H11	O26:H11	Fraser	Water	14	*stx1_*a*_*	β1	294, 379			300
378-O103:H25	O103:H25	Fraser	Water	15	*stx1_*a*_*	θ			L387	
392-O163:NM^d^	O163:H19^*^				*stx1_*a*_ + stx2_*a*_*	NA				

* *Denotes differences between in silico predicted serotype and that assigned by conventional serotyping*.

a *NA, not applicable due to absence of intimin gene*.

b *Multiple partial matches to different stx2 subtypes in genome assemblies*.

c *Number in brackets is days elapsed between recovery of a clustered isolate from the same site*.

d *Reference strain EC19920459 (PHAC-NML, Guelph, Canada)*.

An examination of *stx*/*eaeA* allelic subtypes and additional acquired virulence factor genes (AVFG) with known association to human or animal STEC disease provided further insights on isolate relatedness within clusters. *Stx1, stx2*, and *eaeA* subtypes detected in the sequences are given in Table 5 and AVFG are displayed in the matrix adjacent to the tree in Figure 3. Overall, subtype *stx1*_*a*_ was detected in 33, *stx2*_*a*_ in 16, *stx2*_*d*_ in 6, *stx2*_*b*_ in 3, *stx1*_*c*_in 3 and *stx2*_*c*_ in 2 isolates. The most common *stx* subtype combination was *stx1*_*a*_ alone, found in 20 isolates, followed by *stx1*_*a*_+ *stx2*_*a*_ in 11 isolates. Five allelic variants were detected in the 30 isolates bearing the *eaeA* gene, including β1 (10 isolates), θ (7), ε (7), γ (3), and ζ, (3). Most of the isolates with similar serotypes were in clusters with identical *stx* and *eae* gene subtypes with the exception of 337 and 363, two O8:H19 isolates from different watersheds with discordant *stx* gene subtypes. Seven AVFGs were common to both isolates but 337 lacked the endonuclease colicin E2 *celb* gene, which suggested the isolates were different strains of the same serotype. In contrast, several clustered isolates could not be differentiated by the analyses performed in this study. Some were derived from discrete samples and were likely clonal, for example O103:H2 isolates 389 and 390 recovered from the same sediment sample. However, an isolate of identical serotype (391) but showing evidence of 14 of the 18 AVFGs identified in 389 and 390 was recovered 14 days later from sediment collected in the same site (Table 5). In addition, the three isolates were clustered with a fourth of identical serotype (377) originating from a different watershed but seemingly lacking 2 AVFGs present in 389 and 390. Other instances of serotype recurrence within a sampling site or watershed were evident, including O69:H11 (342, 356), O174:H21 (347, 364), and O5:NM (341, 344). Isolates with similar serotypes were also recovered across watersheds, including three from priority serogroup O157 (371, 375, 293) with identical complements of AVFGs. Additional isolates with comparable complements of AVFGs collected in different watersheds included O26:H11 (294, 379, 300, 301), O111:NM (296, 298), and O111:H8 (297, 299).

Whole genome analyses (Figure 3) also revealed phylogenetic similarities and intergenomic features common to some water/sediment isolates and clinical strains from STEC serogroups or serotypes that cause human disease. For example, O157:H7 isolates 371 and 375 were tightly clustered and assigned AVFG profiles identical to those of clinical strain EDL933, an EHEC isolated from raw hamburger meat implicated in a 1982 outbreak in Michigan (Wells et al., 1983), and strain Sakai associated with a large outbreak caused by contaminated radish sprouts in Japan (Michino et al., 1999). Comparisons within serogroups O26, O103 (including serotypes H2 and H25), O111, and O165 provided additional examples of clustered isolates with virulence gene profiles analogous to those deduced from clinical strain sequences. In contrast, genes associated with aggregative behavior and virulence in enteroaggregative STEC (e.g., *aap, aatA, aggA, aggR, pic*) were not detected in the isolates described in this work. However, isolate 384–O128:H2 appears to bear the recently described virulence plasmid-encoded open reading frames (ORF) 3 and 4 found in serotype O104: H4 prototype strain O42 (Morin et al., 2013).

## Discussion

The LM of British Columbia is characterized by rapid rates of urban development and population growth in a region comprising more than 3000 farms on 90,000 hectares of highly productive agricultural land. Croplands (54,000 ha, 57%) sustain the production of horticultural commodities, notably berry fruit, field and market vegetables. The balance is devoted to pasture and/or building infrastructure to support intensive dairy and poultry production and comparatively smaller hog, sheep, goat, horse and other farm animal herds (Anonymous, 2013; BCMA, 2014). Surface water resources in the LM are exploited intensively for varied agricultural and non-agricultural uses. Growing awareness of the latent risks of human exposure arising from indirect transmission via water mandated a broad assessment of STEC prevalence and characteristics in LM surface waters. The prevalence and diversity of STEC reported here are indicative of recurrent contamination of surface waters in the region. Historical data on STEC prevalence in LM watersheds is limited. The occurrence and sources of *E. coli* O157:H7 over a period of 2 years (2004-2006) were examined in the Salmon River watershed located approximately 20 km north-east of the Serpentine River (see Figure 1). Tracking of *Bacteroides* host-species markers provided evidence that the watershed was affected by multiple potential sources of fecal contamination, including human sewage, specific domestic and wild animal species (Jokinen et al., 2010). Whereas isolation rates for *E. coli* O157:H7 (maximum frequency of 6.7%) using traditional immunomagenetic separation methods were positively correlated with seasonal precipitation, the serotype was not recovered from water during the summer (Jokinen et al., 2010). A seasonal trend was also evident in the LM watersheds examined in the present study, although STEC were isolated from >15% of samples collected during the summer. It must be stressed that prevalence rates reported here were derived from analysis using methodology that improves the sensitivity of detection and isolation of all STEC in water, including serotype O157:H7 (Johnson et al., 2014). Consequently, it is unclear whether discrepancies in the frequency of STEC isolation in the Salmon River watershed and prevalence rates derived from broader geographical samplings in the LM can be ascribed to differences in method performance or to the variable effects of local land use, climate, ecological factors or hydrogeological forces at play in each watershed. Nonetheless, seasonal differences in prevalence and correlation with precipitation events revealed by the present work provide important clues about potential sources of STEC and factors that may affect their dissemination and persistence in surface waters in the region. Higher prevalence rates during wet seasons strongly suggest that hydrological factors likely play an important role in the transport of STEC from land-based sources to surface waters in the LM. Runoff and contaminant loading from manured crops and grassland in the region is known to occur primarily during the wet fall and winter seasons, when rainfall is highest (van Vliet and Derksen, 2003). Moreover, STEC were frequently recovered in a limited number of sediment samples collected from one site examined in the study. While preliminary, this observation combined with the above noted recovery of similar serotypes from the same site at different sampling intervals hints at the possibility of release from sediments caused by turbulent flow during periods of high precipitation (Yakirevich et al., 2013). Clearly, additional research will be required to determine if sediments serve as reservoirs and contribute to the persistence of potentially pathogenic *E. coli* in LM surface waters.

The increasing significance of non-O157 STEC infections has prompted examination of serotypic, phenotypic and genotypic diversity in clinical, animal and food isolates. There have been comparatively few attempts to examine STEC diversity in surface waters. Johnson et al. (2014) isolated 53 STEC serotypes from a major watershed affected by wildlife, agriculture and human activity in Ontario, Canada. Serotyping of isolates from surface waters in the urban-agricultural landscape of the LM returned 33 distinct serotypes, including O157. Isolation of O157 was infrequent, accounting for only 2.7% of all isolates recovered over the course of the study. The scarcity of the serotype was also reported by Johnson et al. (2014) in Ontario waters (4% of all isolates) and by Cooley et al. (2013) in California surface waters where the prevalence of non-O157 isolates was approximately five-fold higher than that of O157. Isolates from other priority serotypes (O26, O103, O111) in the LM presented virulence gene profiles that are frequently reported in clinical isolates, notably those including the *eae* and *stx2* gene (Boerlin et al., 1999). Hence, the observations herein provide additional evidence that surface waters can support highly diversified STEC populations comprising a range of non-O157 serotypes that have been largely overlooked in assessments of potential risks to human health. It is presently not possible to distinguish virulence factors or combinations thereof that reliably predict the potential of STEC to cause human disease (EFSA Panel on Biological Hazards (BIOHAZ), 2013). While the pathogenicity of isolates recovered from surfaces waters in the present study is uncertain, the prevalence of STEC with complex complements of virulence factors present in strains with historical association to human disease is of concern and warrants further scrutiny.

## Conclusions

An improved method of detection and genomic analyses were applied to the examination of STEC prevalence and characteristics in surface waters from four watersheds located in the Lower Mainland of British Columbia, a region impacted by rapid urbanization and intensive agricultural activity. Repeated sampling in the watersheds provided extended preliminary evidence for seasonal variation and geographic differences in the prevalence and diversity of STEC with complex virulence factor profiles known to be associated with human pathotypes. Future assessment of risks to public health caused by non-agricultural and agricultural uses of surface water resources in the region will clearly have to be made in consideration of inherent variation in the spatio-temporal prevalence of potentially pathogenic STEC.

## Author contributions

SN and KA participated in the design of the study, performed sampling, microbiological and molecular analysis of samples, contributed to interpretation of the data, preparation of the manuscript and approval of final version; JC, CL, and VG performed bioinformatic analyses, interpreted the data, contributed to the preparation of the manuscript and approval of final version; RJ and KZ participated in the design of the study, performed serological analyses, contributed to interpretation of the data, preparation of the manuscript and approval of final version; PD, SB, and ET led the design of the study, analyzed and interpreted data, contributed to the preparation of the manuscript and approval of final version.

### Conflict of interest statement

The authors declare that the research was conducted in the absence of any commercial or financial relationships that could be construed as a potential conflict of interest.
